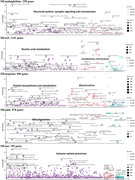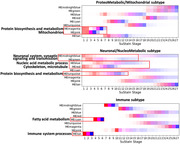# Unraveling temporal transcription dynamics of molecular FTD and ALS subtypes

**DOI:** 10.1002/alz.090423

**Published:** 2025-01-03

**Authors:** Ting Shen, Barbara E Spencer, Vivianna M Van Deerlin, Hemali Phatnani, Eddie B Lee, Corey T McMillan, NYGC ALS Consortium

**Affiliations:** ^1^ Frontotemporal Degeneration Center, Department of Neurology, Perelman School of Medicine, University of Pennsylvania, Philadelphia, PA USA; ^2^ University of Pennsylvania, Philadelphia, PA USA; ^3^ New York Genome Center, New York, NY USA; ^4^ Center for Neurodegenerative Disease Research, University of Pennsylvania, Philadelphia, PA USA

## Abstract

**Background:**

Frontotemporal degeneration (FTD) and amyotrophic lateral sclerosis (ALS) constitute a clinicopathologic spectrum with multifaceted heterogeneities. Brain transcriptomics may help to identify molecular subtypes of FTD and/or ALS but this testing is only possible at autopsy and thus is cross‐sectional and representative of end‐stage disease. Subtype and Stage Inference (SuStaIn) is an unsupervised machine‐learning algorithm that was employed to identify temporal dynamics of data‐driven subtypes of ALS and FTD.

**Method:**

We utilized transcriptomic RNA‐seq data from frontal cortex tissue provided by the NYGC ALS Consortium including individuals with FTD (n = 27), ALS (n = 118), ALS‐FTD (n = 23), and non‐neurological controls (n = 42). After quality control, 19,817 genes were adjusted for age, sex, RIN, contributing site, and cell‐type proportion. Weighted gene co‐expression network analysis (WGCNA) constructed co‐expression modules and the module eigengene of each module was compared between FTD‐ALS and controls. The significantly up‐regulated or down‐regulated modules underwent functional enrichment analyses for biological annotations. We then utilized the gene expression profiles of significant modules to train the SuStaIn model to identify molecular subtypes with distinct gene expression trajectories.

**Result:**

WGCNA identified 16 modules, of which 9 were differentially expressed in case‐control comparisons with functional enrichment in pathways such as “neuronal system”, “nucleic acid metabolism”, “protein biosynthesis and metabolism”, “immune system”, and “mitochondrion” (Fig. 1). The SuStaIn model identified three novel molecular subtypes (Fig. 2): one had early evidence of altered gene expression in “protein biosynthesis and metabolism” and “mitochondrion” (ProteoMetabolic/Mitochondrial subtype); another initially involved pathways related to “neuronal system”, and “nucleic acid metabolism” (Neuronal/NucleoMetabolic subtype); and one subtype had initial involvement of “immune system” (Immune subtype). The ProteoMetabolic/Mitochondrial and Neuronal/NucleoMetabolic subtypes had lower frequencies of ALS compared to the Immune subtype which had a lower frequency of FTD and ALS‐FTD. Moreover, the Immune subtype exhibited a higher frequency of bulbar‐onset and lower frequency of cognitive‐onset than the other two subtypes.

**Conclusion:**

Our results revealed three data‐driven molecular subtypes within the FTD‐ALS spectrum with distinct patterns of early gene pathway involvement. This may contribute to further understanding of potential molecular mechanisms driving some of the heterogeneity and provides the first application of SuStaIn in identifying temporal dynamics of brain transcriptomics.